# Morphological differentiation despite gene flow in an endangered grasshopper

**DOI:** 10.1186/s12862-014-0216-x

**Published:** 2014-10-16

**Authors:** Eddy J Dowle, Mary Morgan-Richards, Steven A Trewick

**Affiliations:** Ecology Group, IAE, Massey University, Private Bag 11222, Palmerston North, 4442 New Zealand; Coastal and Freshwater Group, Cawthron Institute, Nelson, New Zealand

## Abstract

**Background:**

Gene flow is traditionally considered a limitation to speciation because selection is required to counter the homogenising effect of allele exchange. Here we report on two sympatric short-horned grasshoppers species in the South Island of New Zealand; one (*Sigaus australis*) widespread and the other (*Sigaus childi*) a narrow endemic.

**Results:**

Of the 79 putatively neutral markers (mtDNA, microsatellite loci, ITS sequences and RAD-seq SNPs) all but one marker we examined showed extensive allele sharing, and similar or identical allele frequencies in the two species where they co-occur. We found no genetic evidence of deviation from random mating in the region of sympatry. However, analysis of morphological and geometric traits revealed no evidence of morphological introgression.

**Conclusions:**

Based on phenotype the two species are clearly distinct, but their genotypes thus far reveal no divergence. The best explanation for this is that some loci associated with the distinguishing morphological characters are under strong selection, but exchange of neutral loci is occurring freely between the two species. Although it is easier to define species as requiring a barrier between them, a dynamic model that accommodates gene flow is a biologically more reasonable explanation for these grasshoppers.

**Electronic supplementary material:**

The online version of this article (doi:10.1186/s12862-014-0216-x) contains supplementary material, which is available to authorized users.

## Background

Although taxonomy implies abrupt disjunctions between biological entities, we know that speciation usually involves non-instantaneous change [1,2]. The existence of hybrids and the implications of hybridisation have long intrigued evolutionists [3], however the incorporation of gene flow into speciation models has only recently gained acceptance [4-6].

Genetic introgression occurs when two genetically distinct populations come into contact enabling individuals from each to interbreed. When this occurs through secondary contact the process has usually been regarded as hybridisation [7,8], however, broader definitions of hybridisation accommodate the continuum from normal intraspecific mating to rare interspecies exchange [9]. The fertility of resulting offspring mediates gene flow between populations. This situation underpins the popular biological species concept [10], but the frailty and circularity of the *a priori* assumption that species are always reproductively isolated is readily demonstrated [4,11]. Stark reminders that hybridisation is not a valid test of species status come from observations that it is also a potent force in plant speciation that can result in the rapid formation of distinct and reproductively isolated taxa [12,13].

Since the 1960’s, direct measures of variable genetic loci have provided strong evidence that genomes are not unitary and exchange of loci between populations may be uneven [5,9,14,15]. Where gene flow between somewhat distinct genomes is not contained by the formation of hybrid zones [8] or abrupt speciation [16], it can have numerous outcomes; reinforcement of reproductive barriers, the evolution of a new species, the loss of one or both parental species, limited adaption due to homogenization, or provide a means to pass adaptive traits between populations [6-8,17,18].

Gene flow may therefore result in species with mosaic genomes, comprised of alleles from different ancestral populations, which has been described as a potentially important evolutionary mechanism for the formation of many animal species [12,13,15]. Indeed, allelic leakage may be fairly persistent where gene flow is mediated not by extrinsic geophysical barriers, but by locus-specific selection [9,19-23]. Empirical data showing the maintenance of incipient species in the face of ongoing gene flow between populations are gradually accumulating, aided by increasingly sophisticated genetic tools [24-30].

Historically, one of the most informative animal groups in this field of study have been Orthoptera and in particular grasshoppers [14,31-35]. Here we report on flightless New Zealand short-horned grasshoppers (Orthoptera: Acrididae). Most of the fifteen New Zealand species, in four endemic genera, occupy subalpine native grasslands above the tree line [36]. Prior to the arrival of humans in New Zealand (~1260 AD), the landscape was mostly dense forest [37-39]. Grasshopper habitat was therefore mostly in the mountain ranges of the South Island, although a few species occur at lower altitude in areas with semi-arid climate or braided river-beds (*Brachaspis robustus*, *Sigaus minutus* and *Sigaus childi* [40-42]).

**Figure 1:**
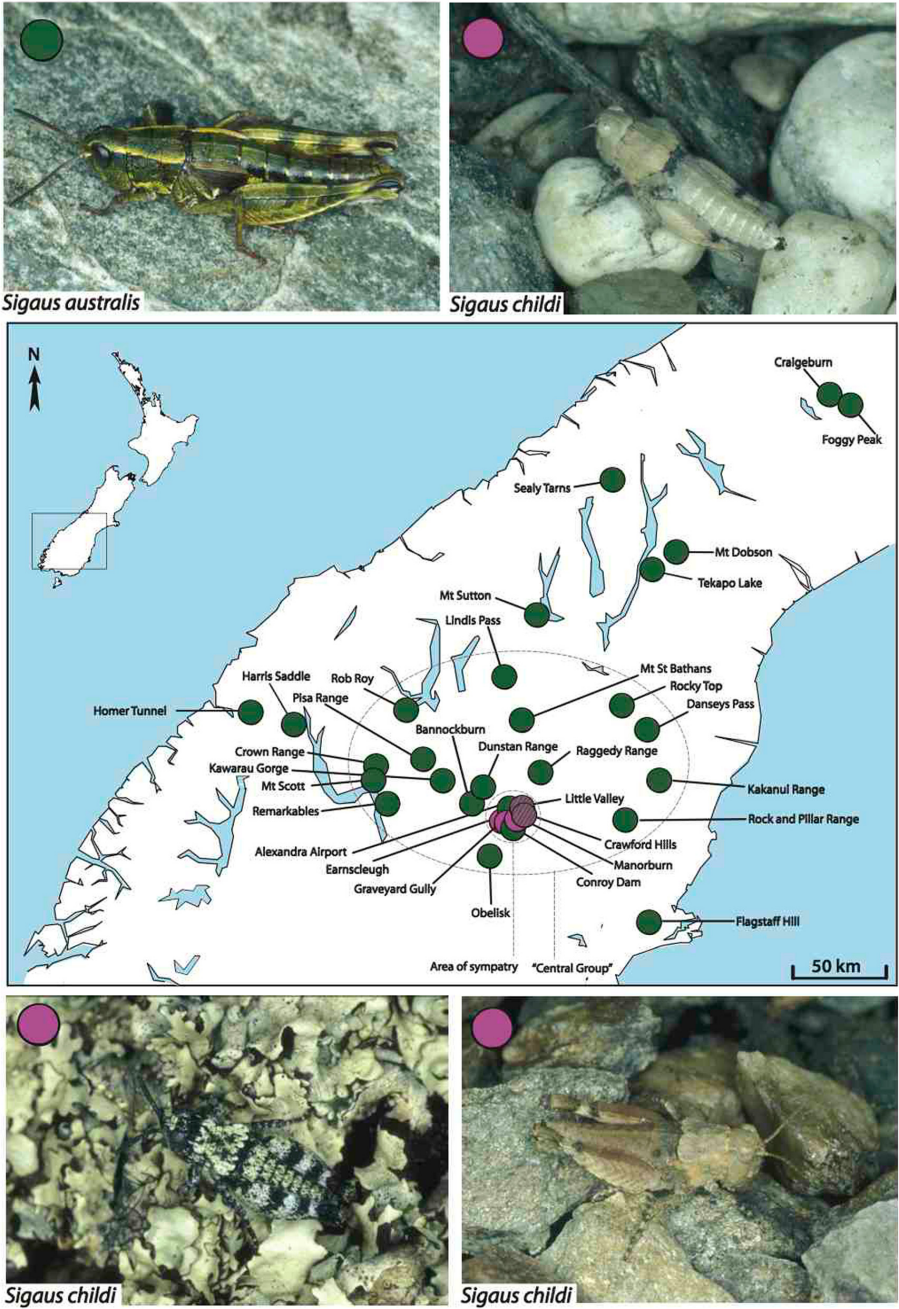
**Sample Map.** Sample locations in South Island, New Zealand, of the *Sigaus australis* complex grasshoppers used in this study. The two main species *Sigaus australis* (green) and *Sigaus childi* (pink) are morphologically very different; *S. childi* tends to be smaller and more camouflaged to its local habitat than *S. australis*. The ‘Central Group’ and ‘Area of sympatry’ defined here are used to analyse subsets of the specimens. Hatched circles represent locations with both species present.

The species *Sigaus australis* appears, on the basis of mtDNA sequence data, to encompass several narrow endemics and one widespread species [42,43]. Typical *Sigaus australis* are relatively large (adult females ~26 mm) and abundant in South Island subalpine grasslands between 1000 and 1800 m asl. Sympatric with this widespread species is the microendemic *Sigaus childi*, which is restricted to a low-lying, semi-arid region of about 100 km^2^ around the town of Alexandra (Central Otago) (Figure 1). *Sigaus australis* is also present in this region, but the two species are readily distinguished by their appearance. An intriguing feature of *S. childi* is that their colour patterns appear to be specific to the substrate on which individuals are found. Colour patterns within *S. childi* range from almost white or grey on quartz pebbles, brown and red on schist gravels, to green and black like the tumbling lichen (*Chondropsis semiviridis*) that grows on rocks in some areas of Central Otago. Although, inferences of camouflage are subjective they support the conjecture that these grasshoppers are under selection by visual predators. *Sigaus australis* are more boldly patterned, often with longitudinal stripes, and tend to be colour-pattern variable within locations.

**Figure 2:**
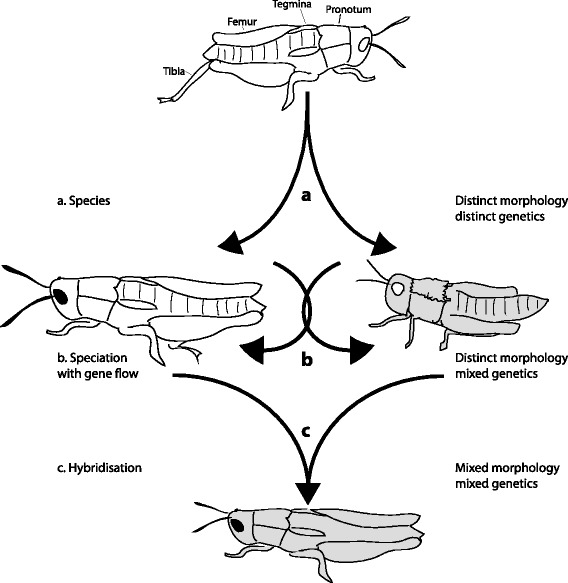
**Alternative hypotheses.** Alternative hypotheses to explain the relationship between morphological differentiation and gene flow in this study of grasshoppers in New Zealand. Two morphologically defined species exist that have a common ancestor and may share identical alleles due to decent, but as distinct species derived alleles are also expected. **(a)** An abrupt speciation event is expected to result in accumulation of distinct traits and genotypes, including at neutral loci. **(b)** Sharing of neutral genetic alleles might be maintained by ongoing gene flow whilst alleles at some loci are subject to selection. Morphological difference is an observable expression of genetic loci under diverging selection. **(c)** Hybridisation or reticulation is expected to result in individuals with intermediate forms.

*Sigaus childi*, although morphologically distinct, could not be distinguished from *S. australis* using mtDNA data [42]. Perhaps *S. childi* evolved recently and has retained ancestral mtDNA haplotypes (Incomplete Lineage Sorting), or has exchanged genetic information since diverging [43]. Or perhaps divergence has occurred and been maintained despite gene flow. Genetic exchange between populations might be experienced at different rates across the genome; selection could operate on some loci to limit local exchange of alleles even when net (genome wide) gene flow continues. These alternatives make different predictions about the pattern of morphological and genetic character sharing (Figure 2)*.* In order to understand the evolution of this system we applied six types of data; morphology, mtDNA sequencing, microsatellite genotyping, multi-copy nuclear sequencing, single nucleotide polymorphisms (SNP) and spatial position. We used these putatively independent data to contrast species integrity as characterised by morphology (subject to natural selection) and neutral characters that allowed us to test the stability of species delimitation, assess the extent and evenness of gene flow and thus gain an understanding of where these grasshopper populations are in the speciation continuum.

## Results

### Morphology

Discriminant analysis of traditional morphological data collected from species description traits of 169 adult grasshoppers revealed strong support for the two entities *Sigaus childi* and *S. australis* (Table 1). All 28 adult *S. childi* were readily separated from the remaining sample containing *S. australis*.

**Table 1:** **Discriminant analysis**

**Summary of classification with cross-validation**		
**True group**	**Predicted group**	
	*S. australis*	***S. childi***
*S. australis*	134	**0**
***S. childi***	**0**	**28**
Total N	134	**28**
N correct	134	**28**
Proportion	1.000	**1.000**

Discriminant analysis with cross-validation using the character states employed in traditional species diagnosis for adult grasshoppers of the *Sigaus australis* complex in South Island, New Zealand. *Sigaus childi* (in bold) individuals were correctly grouped together. The total squared difference between the two groups was 274.

**Figure 3:**
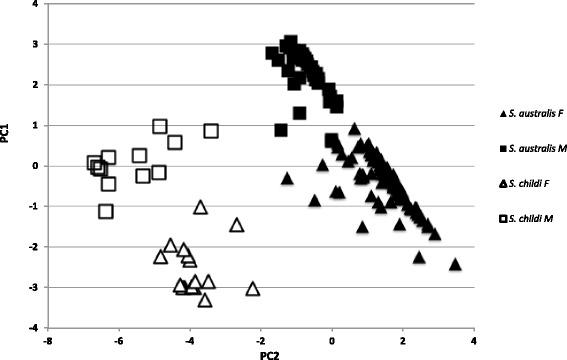
**PCA traditional species diagnostics.** Principle component analysis using morphological character states used in traditional species diagnostics of adult *Sigaus* grasshoppers from South Island New Zealand. Morphologically, *Sigaus childi* is readily separated from *S. australis*, F = females (triangles); M = males (squares).

Principal component analysis also revealed two distinct groups; *S. childi* vs. all other *S. australis* specimens in the analysis with these groups further subdivided into males and females (Figure 3). The first four components of the PCA accounted for >95% of the variation. Thus the morphometric data based on traditional taxonomic characters suggest there are just two morphological entities: *S. childi* and *S. australis.*

**Figure 4:**
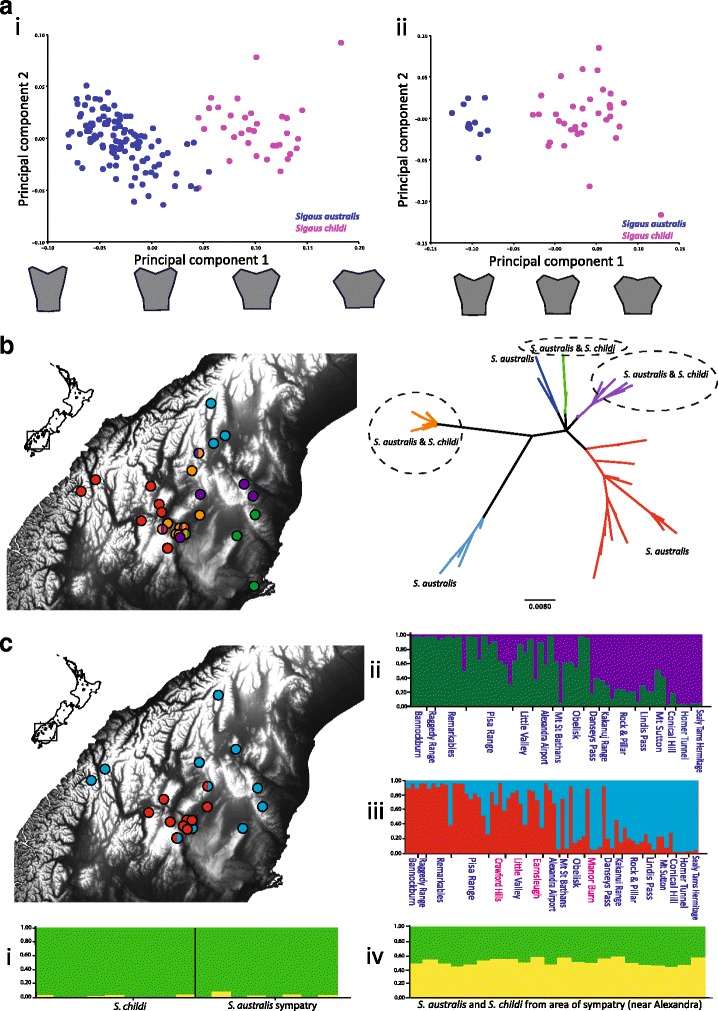
**Genetic and morphological structure within** ***Sigaus*** **grasshoppers in South Island New Zealand. (a)** Variation in the shape of the pronotum using digital imagery of *Sigaus* grasshopper pronotum shape (PCA analysis from MORPHOJ): (i) PCA for both species from all areas, with pronotum shape changes indicated along the PC1 axis. The two major groupings comprise *S. childi* separated from *S. australis* (ii) PCA for just individuals from the area of sympatry (Figure 1), with pronotum shape changes indicated along the PC1 axis. Within the area of sympatry there was no clear evidence of morphological intermediates. **(b)** Neighbour Joining tree of mtDNA haplotypes (COI, 519 bp) and a distribution map showing the spatial distribution of haplogroups. The circled clades (dashed circles) are used in the Network analysis (Figure 5). **(c)** Genetic structure genotype data (STRUCTURE analysis) for the *Sigaus* grasshoppers: (i) Results from the 74 RAD-seq SNPs of the two species in sympatry, *S. australis* and *S. childi* K = 2 (ii) Microsatellite genotypes from *S. australis* populations at K = 2; (iii) Microsatellite genotypes from *S. australis* and *S. childi* individuals (colours shown on map) at K = 2; (iv) Microsatellite genotypes from *S. australis* and *S. childi* individuals from the area of sympatry (see Figure 1) at K = 2.

Geometric analysis of the grasshopper pronotum gave a similar result to that of traditional morphology. PCA analysis of the 14 landmark measurements showed two major groupings (Figure 4a); one composed of *S. australis* and the other composed of *S. childi*. All *S. childi* individuals grouped together due to their distinctive pronotum shape. Variation within *S. childi* was likely due to their extremely cryptic shape formed by the ‘broken’ edge of the pronotum resulting in little uniformity within species. The *S. childi* mean was significantly different from *S. australis* over the entire range (*P* < 0.001 T-square: 1101.9925) and *S. australis* in the area of sympatry (*P* < 0.001 T-square: 935.5225).

The PCA of pronotum shape from those grasshoppers sampled within the area of sympatry was scrutinised for evidence of hybrids (Figure 4a). However, within the area of sympatry, not only did the means of *S. australis* and *S. childi* differ (*P* < 0.001 T-square 1291.4541) but there was no overlap between the two forms. Thus we found no evidence of morphological intermediates in adults or juveniles.

### Mitochondrial DNA sequence

**Figure 5:**
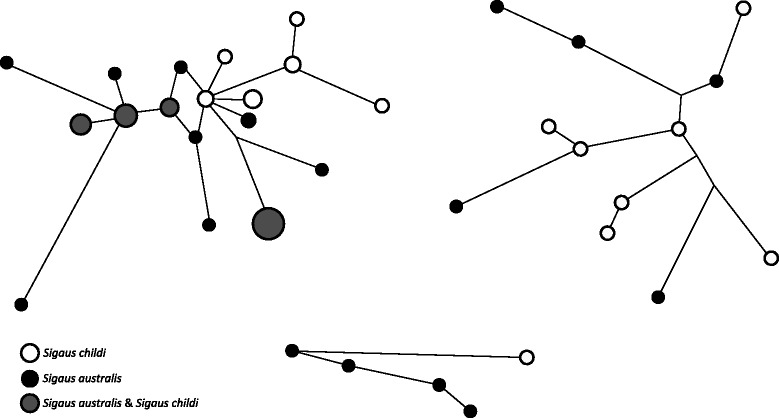
**Minimum spanning networks.** Minimum spanning network of four of the six mtDNA (COI 519 bp) haplogroups (Figure 4a) within the *Sigaus australis* grasshopper complex, show sharing (grey) of identical haplotypes between *S. childi* (white) and *S. australis* (black). Haplotype spot size is proportional to number of individuals with a particular haplotype and branch length estimates nucleotide differences.

Partial Cytochrome oxidase subunit I was sequenced from 59 grasshoppers and combined with 46 previously published sequences [43] (GenBank EF544487–EF544562). A total of 66 haplotypes were identified in the alignment (519 bp) representing 105 individuals (72 *S. australis* and 33 *S. childi*). Six haplotype clusters were identified that were each geographically restricted within the range of *Sigaus australis* (Figure 4b). The MtDNA clusters did not correspond to current taxonomic groups or morphological types. MtDNA sequences from *S. childi* fell in several parts of the *Sigaus australis* complex phylogeny (Figure 4b). NETWORK analysis revealed the extent of sharing between *S. australis* and *S. childi* (Figure 5). As a result of interspecific sharing, some *S. childi* have haplotypes more similar to haplotypes in *S. australis* than other *S. childi*. No single mtDNA clade can be confidently construed as being primarily associated with *S. childi* ancestry. The high genetic diversity detected at Alexandra appears to be a result of the meeting of three distinct mtDNA clades that otherwise have separate ranges.

No evidence for isolation by distance was detected among the mtDNA diversity within the *S. australis* complex (*p =* 0.5410). Although the mtDNA diversity within clades also did not fit a model of isolation by distance there was a non-significant positive relationship (*p =* 0.1410) and the power of this test was limited by smaller within-clade sample sizes. There was no statistical support for population genetic differentiation between *S. childi* and *S. australis* from either the central group F_CT_ = 0.09175 (*P* = 0.09677), or from the area of sympatry F_CT_ = 0 (*P =* 1) (Figure 1).

### Microsatellites

The three microsatellite loci surveyed each had between 16 and 18 alleles. No evidence of linkage disequilibrium was detected and Hardy-Weinberg expectations were met in the majority of population samples. A positive relationship between geographic distance and genetic differentiation (pairwise F_ST_) supported a model of isolation by distance for *Sigaus australis* (Figure 1) (57 individuals; *P* = 0.0108). Analysis of *S. australis* microsatellite data (excluding *S. childi* samples) using STRUCTURE [44] showed evidence of extensive gene flow among populations. The optimum ΔK was K = 2 (Figure 4c), which is consistent with a grouping of populations in Central Otago (Figure 1 and 4b).

Analysis of microsatellite data from 57 *S. australis* and 13 *S. childi* individuals resolved the same geographic subdivision of genetic variation: K = 2 (Figure 4c). There was no support for K = 3, which was unexpected given that the data encompassed two morphologically distinct species and spatial structure had already been indicated. It is important to note that although the microsatellite dataset covers a similar geographical range to that of the mtDNA dataset there is little similarity in the genetic structure detected. To reduce the possible influence of uneven sample size of the two species we restricted the data to include sampling only from the area of sympatry (Figure 1). STRUCTURE [44] analysis found no support for genetic partitioning within these data (i.e. K = 1), contrary to the expectation that the two morphologically defined species would represent discrete genetic units (Figure 4c).

As with the mtDNA data we sought evidence of genetic structure concordant with taxonomy and morphology using analysis of the correlation of genotypes between species by grouping the samples according to the morpho-species *S. australis* and *S. childi* and estimating F_CT._ No significant genetic differentiation between the morpho-species was found within the central group: F_CT_ = 0.01780 (*P =* 0.18573), or the area of sympatry F_CT_ = −0.07424 (*P =* 0.65494), although these samples were not all taken from the same generation*.* This result was consistent with the inference from STRUCTURE.

### Nuclear sequencing

We amplified and sequenced the ITS region (706 bp including 5.8S, ITS1 and 2) from 40 grasshoppers. Some of the grasshoppers had unambiguous single ITS sequence but many had more than one ITS sequence, consistent with these grasshoppers being heterozygotes of mixed ancestry. Of twenty-five grasshoppers (15 *S. childi* and 10, *S. australis*) collected near the township of Alexandra, 16 (11 *S. childi*, 5 *S. australis*) had more than one sequence which differed by the presence of an INDEL approximately 100 bp from the ITS1 forward primer. Sequences of ITS2 from these individuals were unambiguous except at single nucleotide polymorphic sites (SNPs), confirming that these grasshoppers carried more than one ITS sequence per genome. There were 16 SNPs in the set of unambiguous sequences. However the presence of an INDEL near the start of ITS1 meant grasshoppers with more than one sequence had only 13 observable SNPs. Only one of the 15 grasshoppers from outside the Alexandra area (a specimen from Raggedy Range) appeared to have more than one ITS sequence per genome that involved the large INDEL. However, many individuals (mostly collected from the northern part of the species’ range) had an independent 8 bp insertion that occurred in all their copies of ITS. When more than one ITS sequence was detected in a grasshopper DNA, we found that ambiguity could parsimoniously be explained by combinations of unambiguous (single copy) sequences that we separately identified in other grasshoppers. We detected only one copy of ITS in a genome-wide survey of *Sigaus australis*, suggesting that there was only one family of ITS in these grasshoppers; sequence variation within this family occurs where individuals have recently exchanged genetic material.

### Rad-Seq SNPs

The Illumina sequencing provided 9,789,323 forward reads of 100 bp for the 30 grasshoppers, of which 8,934,377 were retained after quality checks in process_radtags.pl (part of the Stacks package). These comprised between 3,743 and 978,246 reads per individual with a total of 30,439 loci. Three individuals (2 *S. australis* and 1 *S. childi*) were removed due to low coverage. From these data sets we identified 8,958 loci that occurred in >2 individuals and these were subjected to further selection based upon coverage per population. The Lindis population was removed from subsequent analysis as several individuals failed to produce data of sufficient quality (likely due to poor DNA quality) and most of the putative-loci resolved were not represented in the other samples. Of 8,958 loci, 74 were retained as they occurred in ≥ 50% of the two populations (*Sigaus childi* and *Sigaus australis* in sympatry). The relatively low proportion of loci that were represented across both population samples was due to insufficient representation of their very large genome despite our protocol involving quanitification and compensation for this. Nevertheless we obtained ample data for our purpose.

**Figure 6:**
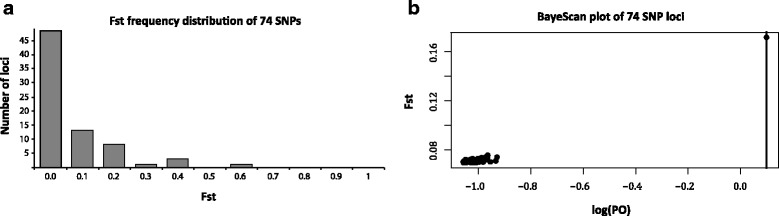
**SNP Loci. (a)** Frequency distribution of locus specific F_ST_ values for each of the 74 SNP loci sampled between the grasshoppers *Sigaus childi* and *Sigaus australis* in sympatry. **(b)** BAYESCAN plot of 74 SNP loci with a single marker (log(PO) >1) showing slight departure from neutrality; the vertical line is the 5% PO threshold of false discovery.

The distribution of pairwise (*S. childi*/*S. australis*) F_ST_ values for each of the 74 putative-loci revealed the high frequency of low scores expected in the absence of significant structure (Figure 6a). A test for deviations from expected frequencies of neutral loci in BayeScan indicated that one marker may have been subject to diversifying selection, log(PO) >0 alpha 0.878 (Figure 6b). A BLAST search of the sequence containing this SNP did not result in any matches to known sequences on Genbank. Mean population pairwise F_ST_ was low (0.025), with a confidence interval that effectively included zero (CI 2.5% 0.001, CI 97.5% 0.053), providing little evidence that these samples represent more than one population, with random mating. Population differentiation estimated with STRUCTURE suggested extensive sharing of genetic material among populations, with no species structure detected (Figure 4ci). This was confirmed by analysis in MIGRATE-N, which indicated extensive gene flow between the species in the zone of sympatry (Table 2).

**Table 2:** **Migration rates**

**All loci**	**Mean**	**0.025**	**0.975**	**Nm**
θ 1	0.09632	0.08413	0.10907	
θ 2	0.08723	0.07547	0.09907	
M2- > 1	2046.7	1927.3	2051.0	49.3 (2- > 1)
M1- > 2	3010.8	2874.7	3010.7	65.6 (1- > 2)

Extensive gene flow between the grasshoppers *Sigaus australis*_1_ and *S. childi*_2_ in sympatry was revealed using MIGRATE-N with 74 RAD-seq SNP makers. Theta θ is an estimate of population size, θ = 4N_e_μ in the SNPs, where N_e_ is population size and μ is mutation rate, population size was generally large as is expected for grasshoppers. Mutation scaled migration rates (M) were converted into Nm (number of migrants per generation) via θ_1_M_2->1_ = 4 Nm_1_. The results show extensive gene flow in both directions.

## Discussion

We found morphological support for two distinct entities consistent with their existing taxonomic treatment as species: *Sigaus australis* and *Sigaus childi. Sigaus australis* has a comparatively wide geographic range that can be subdivided into a number of phylogeographically distinct mtDNA haplogroups. *Sigaus childi* is nested within *S. australis* in terms of habitat, geographic range and genetic diversity; in stark contrast to its clear morphological distinction. Despite occuring in sympatry, no phenotypic intermediates were detected. We found no evidence of genetic partitioning in putatively-neutral mtDNA sequence, microsatellite, ITS sequence loci or SNP data. None of the mtDNA diversity detected within *Sigaus australis* is concordant with current taxonomic subdivision. Microsatellite allele frequencies within *S. childi* are indistinguishable from those within sympatric *S. australis* suggesting recent (and on-going) gene flow. The SNP data show no population structure and extensive gene flow between the two species in sympatry, with one marker showing some sign of diversifying selection. The presence of more than one ITS1-ITS2 sequence within the genomes of single grasshoppers is also consistent with recent gene flow near the township of Alexandra. Concerted evolution normally results in homogenisation of variation in the rRNA cassette containing ITS [45].

A possible explanation for this lack of genetic differentiation is that *S. childi* is a phenotypic variant of *S. australis* generated by different local environmental conditions. Extreme plasticity is known to occur in other grasshoppers such as the locust (*Locusta migratoria*) which has two distinct life history strategies that are partitioned in time [46]. Indeed, *S. childi* is restricted to a limited lowland habitat in contrast with the subalpine environment experienced by most *S. australis*. The lowland conditions of Central Otago have been described as semiarid [47,48], but despite altitudinal separated from subalpine areas, these habitats are climatically similar in terms of their extreme day/night and seasonal temperature cycles. More pertinent is the fact that *S. australis* and *S. childi* are sympatric in the lowland semiarid environment of Central Otago. They occur at the same places at the same times with, for example, specimens of both morphotypes used in our analysis collected as adults within metres of each other in Little Valley, Alexandra on the same day. These circumstances are inconsistent with an interpretation of phenotypic differences being driven by environmental induced plasticity. Although we cannot exclude this possibility, a novel type of micro-environmental control of grasshopper development would need to be invoked.

Alternatively, the observed morphological divergence but lack of genetic structure is consistent with strong character specific selection in the presence of high levels of gene flow or incipient speciation (Figure 2b). Under both such circumstances neutral genetic markers, such as those examined, may not detect population structuring [22]. Contemporary introgression can be difficult to distinguish from incomplete lineage sorting [49,50], however the sharing of identical mtDNA haplotypes and sharing of alleles across neutral nuclear loci in these two species suggests that very recent and/or ongoing reticulation is more likely. Gene flow is expected to homogenise variation between species and it has traditionally been considered that speciation is unlikely to proceed in the presence of gene flow. Many models of speciation have emphasized partitioning of populations by some extrinsic process (e.g. allopatry) as a prerequisite [10,51]. However, if selection on particular loci is sufficiently intense, the effects of gene flow could be mitigated. Models that accommodate permeability of putative species boundaries and acknowledge that selection can be locus-specific rather than genome wide are not new [5,14,20,52], and empirical data that demonstrate this process are emerging [30,53-55].

Different evolutionary responses to selection could explain the observed morphological distinction. *Sigaus childi*, is a small, highly cryptic species of grasshopper suggesting it has been or is under selection from visual predators. In contrast, *Sigaus australis*, is a larger grasshopper, usually with more striking colour markings that are more easily observed against the substrate, suggesting a different mode of predator avoidance. Although the two species appear to share the same neutral alleles; they remain morphologically distinct with a single genetic marker showing some evidence of diversifying selection between the two species. All individuals examined fell into one of two morphological groups and no specimens could be classed as morphologically intermediate within the area of sympatry even when juveniles were examined. The apparent lack of morphological intermediates (hybrids) suggests that selection, even in today’s highly modified environment, is intense. It should be noted, however, that F1 phenotypes are often not intermediate between parentals and this might also explain our observations [56]. The conservation status of *S. childi* limited our sample sizes and precluded any observation of mating behaviour and juvenile colouration and survival where the species are sympatric. Further examination might reveal morphological intermediates.

Situations where speciation and selection are most likely to be observable in nature are those with high environmental heterogeneity, temporal instability and/or novel environments [57,58]. Not surprisingly many examples of contemporary speciation in action therefore come from anthropogenic settings [59,60], and this may be relevant to these *Sigaus* grasshoppers. The South Island of New Zealand was settled by Polynesian colonists starting about 800 years ago, and this was accompanied by episodes of scrub and forest fire [61,62]. Expansion of grass and herbs following reduction of forest that may previously have formed a habitat barrier between the alpine and lowland grasshoppers may have facilitated population mixing. The area now shared by *S. childi* and *S. australis* was further modified by European introduction of plants and grazing animals, and mining practices in the last 150 years. These changes could have facilitated increased gene flow, but were analogous to the effects of Pleistocene climate cycling. Disentangling their respective influence on the grasshoppers is not simple [63].

The taxonomic status of these species is problematic, as traditional methods cannot resolve the conflicting information from morphology and genetics resulting from the process of evolution. Although *S. childi* is not genetically isolated from *S. australis* it is morphologically well differentiated, and in our relatively small SNP dataset we were able to find one marker possibly under selection. Models of speciation with gene-flow predict a continuum from partially isolated populations to reproductive isolation [5,23]. In the *Sigaus* system divergence seems to be at an early stage; *Sigaus australis* and *Sigaus childi* do not appear to be losing morphological distinction, but our neutral genetic data does show extensive gene flow. The absence of any morphological hybrids suggests selection is intense, removing relatively conspicuous intermediates and holding these two species apart. This may provide them the opportunity to diverge at other loci.

## Conclusions

The findings of our study endorse Charles Darwin’s original dynamic view of speciation [3], but are contrary to those expected from a more restrictive but popular view of species as reproductively isolated units [10]. There is a grand irony that while for many, genetic methods are seen as tools for testing species status (e.g. DNA barcoding), genetic data are actually the key to revealing that speciation is not clear cut [2,20,23,54,55]. In our study we found that the only characters that reliably distinguished species were morphological, whilst 78 neutral genetic markers showed that distinct morphotypes do not correspond to genetically isolated units.

## Methods

We collected grasshoppers by hand when they were active during the New Zealand summer season (December-March, between 1995 and 2009). Sampling included all recognised members of the *S. australis* complex (*S. australis*, *S. childi*, *S. obelisci* and *S. homerensis*) from their full geographic range (Figure 1). As already noted *S. australis* is widespread in subalpine habitat with a few populations extending down to low elevation (~300 m asl) areas in some locations. *Sigaus childi* occurs only at a single low elevation site in Central Otago where it is sympatric with *S. australis*. Due to the legal protection given to the endangered *S. childi*, sample sizes were limited and sampling spanned more than one overlapping generation (c.f. usual assumptions of population genetic models). Sampling from different generations, which are already overlapping is, however, not likely to increase the similarity of population allele frequencies, and therefore we do not consider this will have hindered any of the analyses. *Sigaus obelisci* and *S. homerensis* are each recorded from single subalpine locations within the range of *S. australis*. The identity of *S. obelisci* and *S. homerensis* specimens were confirmed by Simon Morris (pers comm. to SAT). Individuals were preserved by freezing or in 95% ethanol and identified following Bigelow [36], Morris [64], and Jamieson [40].

### Morphology

Morphological data were collected for all adult *Sigaus australis* complex grasshoppers in two ways. The first used the traditional species diagnostic characteristics, although we note that much of the information used to distinguish some of these taxa has been geographic location and altitude [65,66]. The exception is *Sigaus childi*, for which the sinuous caudal margin of the pronotum and tegminal size are diagnostic [40]. Male genitalia are taxonomically informative for some grasshopper species but consistent differences have not been reported among species in this complex. For instance, male genitalia in *S. homerensis* and *S. obelisci*, are each described as being near identical to *S. australis* and *S.* “remarkables”*,* which is a synonym of *S. australis* [64-66]. Thus male genitalia can be interpreted as being variable within *S. australis,* but uninformative for species delimitation [36,40,64]. Because of this absence of diagnostic information and in light of previous genetic information [43] *S. homerensis* and *S. obelisci* are incorporated into *S. australis* here.

The traditional species diagnostic characters for the grasshoppers were examined and measured with the aid of a dissecting microscope. Four metrics were recorded for each grasshopper using callipers accurate to 0.01 mm; maximum pronotum width, mid-line pronotum length, femur length and body length. In addition, six characters with discrete states were examined; sex, length of tegmina (see below), shape of pronotum posterior margin (sinuous or concave), cuticle rugosity (rugose or smooth), shading on pronotum posterior margin (pigmented or not), and shape of pronotum lateral margins (irregular or smooth). For most characters, the alternative and intermediate states were coded as 2, 0 or 1 respectively. The length of tegmina was classified by reference to the number of abdominal tergites across which they extended; not beyond the pronotum (as in many *S. childi*) coded 0, not beyond first abdomen segment coded as 1, and so forth to a maximum of 4 (no tegmina ever reached beyond the posterior margin of the 5th tergite).

Adults were distinguished by the tegmina concealing the relictual hind wing, which is the case only in the last instar. Juveniles were excluded from this morphometric analysis. The data were analysed using Discriminant Analysis and Principle component analysis (PCA) approaches implemented in MINITAB 15 [67]. The discriminant analysis with cross validation tested whether character information could be used to group individuals into their *a priori* categories: *S. australis* and *S. childi*. A PCA was applied to all the morphological characters and the scores saved. PCA required no *a priori* grouping, allowing us to determine whether the data could be partitioned into taxonomically meaningful groups based solely on the documented morphological character states.

As an alternative to the traditional taxonomic characters, we tested for shape differences of the pronotum among species using geometric analysis. This method is more powerful and avoids any circularity that could arise from using traditional species characteristics as the sole morphological traits analysed. Much of the taxonomy in the *Sigaus* genus relies on the pronotum shape, but descriptions are often vague, based on discrete states and inferred from few individuals making species identification difficult [36,65,66]. Using two digital images of the pronotum of each of 147 individuals (113 *S. australis*, 34 *S. childi*) that were obtained with the aid of a dissecting microscope we tested whether shape variation could be detected from metric data. Using IMAGEJ [68], 14 landmarks were identified around the perimeter of the dorsal surface of the pronotum on each image of each grasshopper and measured. The landmarks were selected to maximise variation among individuals. These measurements were analysed using MORPHOJ [69]. A procrustes fit aligned by principal axes was performed to eliminate size differences before a Procrustes ANOVA was used to examine the error of image capture. This analysis revealed that the error arising from image capture variation was biologically irrelevant: mean squares for image capture was 32 times smaller than the variation found between individual grasshoppers. Juveniles, adults and both sexes were included in the analyses and tested to confirm they did not partition in the results. Principal component and discriminant analyses with cross validation were each preformed on the averaged value for each individual from all four species (34 *S. childi* and 113 *S. australis*). These analyses were also separately applied to the *S. childi* and *S. australis* individuals collected within the area of sympatry (34 *S. childi* 12 *S. australis*) (Figure 1).

### Mitochondrial DNA sequence

In order to improve genealogical resolution in relation to taxonomy and geography, we supplemented existing published (genbank EF544523-EF544562) mtDNA Cytochrome Oxidase Subunit I (COI) data for the *Sigaus australis* grasshopper complex [43]. Tissue was dissected from femora of recently collected grasshoppers and DNA extracted using a salting-out method [70,71]. DNA from specimens preserved for more than one year, was extracted using incubation at 55°C with Proteinase K and a CTAB buffer (2% Hexadecyltrimethylammonium bromide, 100 mmol/L Tris–HCl pH8.0, 1.4 mol/L NaCl, 20 mmol/L EDTA), followed by a combined phenol/chloroform/isoamyl alcohol (25:24:1) cleanup. Extractions were eluted in water and diluted as necessary for PCR reactions. Primers C1-J-2195 and LI-N-3014 [72] were used to target the 3' portion of COI. Polymerase chain reactions (PCRs) were performed in 10 μl volumes using ABgene Red Hot Taq (Thermo Fisher Scientific). Thermocycling conditions were 94°C for three minutes; 94°C for 45 seconds, 52°C for 45 seconds and 72°C for 75 seconds repeated 36 times; followed by a 2 minute final extension. Cycle sequencing used Perkin Elmer BigDye 3.1 chemistry following the manufacturer's protocols analysed on an ABI Prism 377 DNA sequencer (Applied Biosystems, Inc., Foster City, California). Sequences were checked using SEQUENCHER version 4.10.1 (Gene Codes) and aligned with existing data using SeAl version 2.0 and GENEIOUS PRO version 5.3.4 [73,74].

GENEIOUS was used to estimate a neighbour-joining tree for all the lineages. NETWORK version 4.5.1.6. [75] was used to estimate haplotype networks within clades. To test for a correlation between genetic and geographic distance (expected under a model of isolation by distance), Mantel tests [76] were performed using ISOLATION BY DISTANCE WEB SERVICE version 3.16 [77] with 10,000 randomizations to assess the significance of distance correlations. Distance by distance analysis was applied to all data and separately to data within haplo-groups.

A standard AMOVA was used to test for significant genetic differences based on the estimate of genetic partitioning among groups (F_CT_) using ARLEQUIN version 3.5.1.2 [78]. The first run tested *S. childi* against all populations in the central group (as indicated in Figure 1) and the second included only those individuals from the area of sympatry (Figure 1).

### Microsatellites

To examine population structure using nuclear loci we developed primers to amplify microsatellite loci using a modified enriched microsatellite library protocol (Additional file 1).

Screening of fifty microsatellite loci revealed three that were polymorphic and amplified consistently among a subset of DNA samples from the target taxa. The loci were checked for large allele dropout, stuttering, and null alleles using 1000 randomisations in MICROCHECKER version 2.2.3 [79]. Not all populations had sufficient sampling to analyse in MICROCHECKER and within the area of sympatry the *S. childi* and *S. australis* populations were treated as a single population for this purpose. Although there was evidence of null alleles in some of the loci within some of the populations (*S. childi* sympatry and *S. australis* sympatry), this is unlikely to influence the detection of genetic differentiation [80]. To test a hypothesis of isolation by distance, geographical distances (km) among pairs of *S. australis* population samples were linearly regressed against their pairwise F_ST_ estimates. Nineteen populations had sufficient sampling for this analysis (Figure 1). Mantel testing [76] was performed using ISOLATION BY DISTANCE WEB SERVICE version 3.16 [77] with 10,000 randomizations to assess the significance of distance correlations.

Population structure was assessed without *a priori* groupings using STRUCTURE version 2.3.4 [44]. First we looked for evidence of population structure in the data from *S. australis* samples only, secondly among all samples from all areas sampled for the complex, and thirdly among all samples collected in the area of sympatry (*S. childi* and *S. australis*) and central group *S. australis* (Figure 1). The analyses were run using an admixture model with correlated allele frequency, 100,000 generations of burn-in followed by 100,000 generations, and the number of groups (K) set from 1 to 20 (10 replicates each). The optimum value of K was found using the ΔK method except for K = 1, which was determined by examination of the bar-plots and structure harvester [81]. Charts were averaged over the 10 replicates and re-drawn using CLUMPP and distruct [82,83]. We sought evidence of genetic differentiation concordant with morphology using the populations within the central group subset identified by STRUCTURE (Figure 1).

A standard AMOVA was used to test for significant genetic differences based on the estimate of genetic partitioning among groups (F_CT_) using ARLEQUIN version 3.5.1.2 [78]. The first run tested *S. childi* against all populations in the central group and the second only those *S. childi* and *S. australis* individuals from the area of sympatry (Figure 1).

### Nuclear sequence

Nuclear sequences representing the internal transcribed spacers (ITS1 and ITS2) of the rRNA cluster and the intervening rRNA 5.8S gene were obtained using the primers ITS4 and ITS5 [84]. PCR conditions and sequencing followed standard protocols as above. Sequences were aligned using GENEIOUS PRO version 5.3.4 [74] and checked by eye. Sequences were generated for all grasshoppers from the area of sympatry of *S. childi* and *S. australis* (the Alexandra region). Alignment and comparison of unambiguous with ambiguous sequences allowed us to identify the most likely combinations of sequences that gave the observed heterozygotes (S3). Where sequence variants differed by single nucleotide substitutions we could identify and resolve the polymorphism. Where sequence variation involved INDELs the resulting length polymorphism was evident by abrupt onset of sustained nucleotide ambiguity at the INDEL position, but sequencing in both directions allowed identification of the combination of sequences involved. To examine the number of families of ITS per grasshopper genome we interrogated a DNA dataset generated by high throughput sequencing. Genomic DNA from a single *Sigaus australis* individual was sequenced on an Illumina Hi-Seq 2000 (Beijing Genomics Institute) resulting in >1GB of sequence. The sequence was de-novo assembled via VELVET [85] with mapping performed using BOWTIE version 2 [86] and the results viewed in TABLET version 1.12.09.03 [87]. The resulting contigs were blasted to Genbank and all matches to ITS were selected, aligned, mapped back and, checked for copy number.

### Rad-Seq SNPs

Single nucleotide polymorphic (SNP) anonymous nuclear markers were generated using high throughput sequencing, with individual DNA fragments coded so we could identify individual grasshopper genotypes. The double digest Rad-Seq protocol [88] was applied with minor modifications. We estimated genome size to help us optimise the selection of endonucleases and sequencing coverage. To do this we used flow-cytometry on a FACSCalibur system and CellQuest software (BD Biosciences, San Jose, CA, USA), following staining of cells with Propidium Iodide and reference to an internal control (chicken or locust). Our estimates of the *Sigaus* genome were approximately 11.9 pg (consistent with estimates of other short-horned grasshopper species (http://www.genomesize.com)). In light of this information we used the restriction enzymes PstI and BamHI to digest the whole genomic DNA extracted from 30 grasshoppers (10 *S. childi*, 10 *S. australis* from Alexandra, 10 *S. australis* from Lindis). The DNA fragments were tagged with DNA sequences that identified each individual before size selecting at 300-400 bp and pooling as per [84].

Data were generated using an Illumina Hi-Seq (New Zealand Genomics Limited), and sorted using the STACKS version 0.99992 pipeline [89]. Settings for coverage and sites per read were adjusted iteratively. Read coverage settings vary in the literature [88], so we initially ran trials ranging from 7 to 30 reads, but found no alteration in the results. We report results using an optimum coverage of 15 reads per individual (excluding all stacks with a lower coverage), a maximum of two mismatches between reads for a single individual as well as allowing four mismatches between primary and secondary reads within ustacks. We allowed the program to remove any potentially spurious highly repetitive stacks. In cstacks we allowed 3 mismatches between samples when generating the SNP set (−m 15 -N2 –M4 –n3 -t). We restricted our analysis to a single SNP per putative locus (always the first), thus avoiding potential problems of non-independence between markers. Data file conversion for programs was performed using PGDSPIDER version 2.0.4.0 [90]. Population pairwise F_ST_ as calculated for each putative-locus across all loci in STACKS, and an AMOVA was run in GENODIVE version 2.0b24 [91] to determine F_ST_ across populations. STRUCTURE version 2.3.4 [44] was used to estimate population differentiation using an admixture model with correlated allele frequency. A burnin of 100,000 generations was followed by 10 replications of 100,000 generations with the number of groups (K) set from 1 to 3. The optimum value of K was found from ΔK method, via structure harvester, except for K = 1, which was determined by examination of the bar-plots [81]. Charts were averaged over the 10 replicates and re-drawn using CLUMPP and distruct [82,83]. STRUCTURE was run using one SNP per putative-locus (read), and each putative-locus appeared in both *S. australis* and *S. childi* populations, and occured in ≥ 50% of the individuals.

Gene flow between the two populations in sympatry was estimated using MIGRATE-N version 3.5.1 [92,93], although algorithms that test for gene flow are often not ideal for situations where gene flow is very high, which is likely in this case. MIGRATE-N was implemented with the Bayesian inference strategy. Initial runs involved only half the markers as we optimised settings. The starting values for θ and M were generated initially from F_ST_ with subsequent runs using the resulting θ and M values. The uniform prior distributions were used for both parameters with slice sampling; one long chain was run recording every 5 steps after a burnin of 50,000 with a static heating scheme with five chains. Four runs were conducted on half the data before three final runs on all loci were undertaken using the starting values for θ and M of the previous run. BAYESCAN version 2.01 [90,94,95] was used to examine the individual markers for evidence of selection using the default settings. Prior odds of a neutral model were 10 times more likely than the model with selection at a locus. This prior was tested further by changing it to one, without any identifiable change in results. The alpha value was used to determine the direction of selection with a positive value suggesting diversifying selection and a negative value suggesting balancing selection. Results were viewed in R version 3.0.0 using an FDR of 0.05 [96] and markers with evidence of selection were subjected to a BLAST search via NCBI [97].

### Data archiving

All sanger sequenced data (KM576255-KM576292) and raw Illumina reads (bioproject-261083 http://www.ncbi.nlm.nih.gov/bioproject/261083) are available on genbank. ITS sequence data from *de novo* assembly is available on genbank (KM576254). ITS data indicating SNP and indel variation is available in Additional file 1. Data used in the geometric morphometrics analysis is provided in Additional file 2 along with the SNP/microsatellite set used in this paper (Additional file 3).

### Ethics

This study did not require ethical approval.

## Additional files

Additional file 1:
**Extended Methods.** Further details on methodology. Includes details on the microsatellite discovery methodology, along with primer sequences. As well as details on sample number/location at each analysis. As well as a table indicating SNP and indel variations/positions in ITS sequencing. Additional file 2:
**Geometric Morphometrics.** Procrustes fit values for geometric morphometrics. Additional file 3:
**SNP and microsatellites.** Spreadsheet of SNPS and microsatellites values across populations used in this study.
