# Supplementary material for: Morphological differentiation despite gene flow in an endangered grasshopper
Source: BMC Evol Biol. 2014 Oct 16;14:216. doi: 10.1186/s12862-014-0216-x (PMC4219001; doi:10.1186/s12862-014-0216-x)
Supplement: Additional file 1: — Extended Methods. Further details on methodology. Includes details on the microsatellite discovery methodology, along with primer sequences. As well as details on sample number/location at each analysis. As well as a table indicating SNP and indel variations/positions in ITS sequencing. [file 12862_2014_216_MOESM1_ESM.docx]

**Supplementary 1Microsatellites**

To obtain neutral nuclear loci to examine population structure, we developed primers to amplify microsatellite loci using a modified enriched microsatellite library protocol (Vaughan Symonds protocol per. comm.). Total genomic DNA was extracted from the hind femur of a *Sigaus childi* grasshopper from Manor Burn near Alexandra, via the CTAB method (see mtDNA methods). The DNA was digested using BfuCl (4U/µL) before being ligated to Sau3AI (BfuCI) linker (25 µL). Successful ligation was ensured by amplification with Sau L-A oligo (20µL). 12 cycles of a standard PCR protocol were used for hybridisation. A linker created using 10X T4 Ligase buffer, Sau L-B oligo (100 µL) and polynucleotide kinase, was annealed using 10X ligase buffer, NaCl (600 mM) and Sau L-A oligo (100 µL). Hybridisation used 50-biotin-labelled (CA12 or GA12) oligonucleotides (100 µM), the linker primer Sau L-B (50 µ L) and HYB-A buffer (12x SCC/0.2% SDS) solution (heated to 65ºC to re-suspend). The hybridisation protocol was a gradual step-down cycle from 71ºC to 52.5ºC stopping every 0.5 ºC for 1 min after the PCR product was denatured at 95ºC for 3 min. Following hybridisation, stringency washes with biotinylated beads (Promega) were used to enrich the sample. The biotinylated beads were prepared by washing in HYB-B buffer (6x SCC/0.1% SDS). The following washes were performed, with beads re-captured between steps using a magnetic stand: HYB-C buffer (3x SCC/0.1% SDS) at room temperature (twice), HYB-D buffer (1x SCC/0.1% SDS) at 42ºC for 15 min followed by HYB-D buffer at 60ºC for 15 min, HYB-D buffer at 52 ºC for 15 min. After enrichment the DNA was removed from the beads using TE buffer (10mM tris (HCl) pH 7.5) at 95ºC for 5 min. The resulting DNA was transformed into competent Escherichia coli cells via a vector using the TOPO cloning kit (Invitrogen).

The *E. coli* cells were plated and cultured at 37°C overnight before 768 recombinant colonies were picked, and suspended in 20µL of broth (tryptone, yeast extract and NaCl) and incubated overnight. The inserts were then amplified using M13 primers. A total of 384 colonies were PCR screened, half had been hybridised with the CA12 probe and half with a GA12. We designed primer pairs to target 96 putative microsatellite loci using the programme Primer3 ([Rozen and Skaletsky 2000](#_ENREF_1)). The forward primer of each pair was synthesied with an M13 tail to allow incorporation of a florescent label. Primers were tested using DNA from four different New Zealand grasshoppers; two *S. australis*, one *S. childi* and one *Brachaspis robustus* (Acrididae). PCR amplification was performed in 10µl volumes using ABgene Red Hot Taq (Thermo FisherScientific) using the following protocol: 94°C for three min; 12 cycles of 94°C for 45 s, 62°C for 45 s and 72°C for 45 s; 24 cycles of 94ºC for 45 s, 58ºC for 45 s and 72ºC for 45 s; followed by 10 min at 72ºC. Subsequently fifty putative microsatellites loci were amplified as above using M13 tagged dyes 6FAM and HEX (Invitrogen and Applied Biosystems). Successful amplification was checked by electrophoresis. Loci that gave good amplification were pooled and genotyped on an ABI 3730 with an internal LIZ -250 size standard and scored using Genemapper v3.7 (Applied Biosystems).

**Table S1**

Example sequences for the three microsatellite loci, with the size range of alleles and number of alleles found at each in our sample.

| Micro | Seq | Size range | Number of alleles |
| --- | --- | --- | --- |
| GA31 | \| GACGTTGTAAAACGACGGCC**CCTATCGGTGGGACGTTTTA** \| \| --- \| \| **CCTATCGGTGGGACGTTTTA** \| | 249-284 | 18 |
| C36 | \| GACGTTGTAAAACGACGGCC**CGAATGTACCATTGCTCTGTG** \| \| --- \| \| **ACATCACTACCTCGCCAACC** \| | 166-213 | 18 |
| C44 | \| GACGTTGTAAAACGACGGCC**GATGGCTGCAGTTCTGTTCA** \| \| --- \| \| **CCAGTGGTGGCAGTGTGTTA** \| | 216-248 | 16 |

**Table S2**

The sites, species and number of individuals used in each of the analysis. Microsatellite (Micro), Cytochrome oxidase subunit I mitochondrial DNA sequencing (mtDNA), traditional diagnostic morphological characters (Trad. Morph.), morphometric data subject to geometric analysis (Geo. Morph), intragenic transcribed spacer DNA sequence (ITS) and RADSeq SNPs (Rad Seq).

|  | |  | Micro | mtDNA | Trad.  morph. | Geo.  Morph. | ITS | RADSeq |
| --- | --- | --- | --- | --- | --- | --- | --- | --- |
| Bannockburn | *S. australis* | | 3 | 8 | 2 | 6 |  |  |
| Raggedy Range | *S. australis* | | 2 | 2 | 1 | 2 | 1 |  |
| Remarkables | *S. australis* | | 6 | 6 | 20 | 17 | 2 |  |
| Pisa Range | *S. australis* | | 9 | 6 |  | 3 |  |  |
| Little valley | *S. australis* | | 4 | 5 | 4 | 3 | 2 |  |
|  | *S. childi* | | 2 | 4 | 5 | 5 | 3 |  |
| Alexandra Airport | *S. australis* | | 4 | 6 | 3 | 5 | 6 | 10 |
| Mt St Bathans | *S. australis* | | 2 | 3 | 4 | 6 |  |  |
| Obelisk | *S. obelisci (S. australis)* | | 4 | 3 | 3 | 21 | 3 |  |
| Danseys Pass | *S. australis* | | 3 | 3 | 17 | 1 |  |  |
| Kakanui Ranges | *S. australis* | | 2 | 1 | 3 | 2 | 1 |  |
| Rock and Pillars | *S. australis* | | 5 | 2 | 23 | 7 | 1 |  |
| Lindis | *S. australis* | | 3 | 4 | 2 | 18 | 2 |  |
| Mount Sutton | *S. australis* | | 3 | 3 | 8 |  | 1 |  |
| Harris Saddle | *S. australis* | | 2 | 2 | 9 |  |  |  |
| Homer Tunnel | *S. homerensis (S. australis)* | | 3 | 3 | 3 | 3 |  |  |
| Sealy Tarns | *S. australis* | | 2 | 2 | 4 | 2 | 2 |  |
| Crawford Hills | *S. childi* | | 3 | 5 | 5 | 7 | 3 |  |
|  | *S. australis* | |  | 2 | 1 |  | 1 |  |
| Earnscleugh | *S. childi* | | 4 | 9 | 6 | 9 | 3 | 10 |
| Manor Burn | *S. childi* | | 4 | 8 | 9 | 8 | 4 |  |
| Graveyard Gully | *S. childi* | |  | 7 | 3 | 7 | 2 |  |
| Conroys Dam | *S. australis* | |  | 2 | 4 | 2 | 1 |  |
| Mt Dobson | *S. australis* | |  | 3 | 7 |  | 1 |  |
| Rocky Top | *S. australis* | |  | 1 | 5 |  |  |  |
| Flagstaff | *S. australis* | |  | 1 | 7 |  |  |  |
| Tekapo | *S. australis* | |  | 1 | 1 | 7 |  |  |
| Dunstan | *S. australis* | |  | 1 | 1 | 1 | 1 |  |
| Robroy | *S. australis* | |  | 1 |  |  |  |  |
| Kawarau | *S. australis* | |  | 1 | 1 | 1 |  |  |
| Craigeburn | *S. australis* | |  |  | 1 | 1 |  |  |
| Crown Range | *S. australis* | |  |  |  | 2 |  |  |
| Foggy Peak | *S. australis* | |  |  | 3 | 1 |  |  |
| Mt Scott | *S. australis* | |  |  | 3 |  |  |  |
| Total |  | | 70 | 105 | 168 | 149 | 40 | 30 |

**Supplementary 2 ITS**

**Table S3**

ITS sequence variation in *Sigaus australis* complex grasshoppers. Only polymorphic sites from comparison of all sequences are shown. Nucleotide variable sites and INDELs are shown as P1, P2 etc and I1, I2 etc respectively in the header. Those individuals showing nucleotide site ambiguity and sequence asynchrony following INDEL 3 are likely to contain more than one ITS sequence variant. Sequence asynchrony most probably results from the presence of an INDEL in one ITS variant resulting in complete ambiguity downstream, in otherwise “clean” sequences.

| Species | N | Area | P1 | P2 | P4 | I2 | I3 | P5 | I7 | P6 | I8 | P7 | P8 | I9 | P9 | P10 | P11 | P12 | P13 | P14 |
| --- | --- | --- | --- | --- | --- | --- | --- | --- | --- | --- | --- | --- | --- | --- | --- | --- | --- | --- | --- | --- |
| *S. australis* | 1 | Rock and Pillar | A | A | C |  | 3 | C |  | G | 9 | C | A |  | C | G | T | A | C | A |
| *S. australis* | 2 | Little Valley/Crawford | A | A | C |  | 3 | C | 2 | G |  | C | A |  | C | G | T | A | C | A |
| *S. childi* | 1 | Little Valley | A | A | C |  | 3 | C |  | G | 9 | C | A |  | C | G | A | A | C | A |
| *S. australis* | 4 | Sealy Tarns/Kakanui Ranges/Sealy Tarns/Mt Dobson | G | A | C | 8 |  | C |  | G |  | C | A |  | C | A | A | C | C | C |
| *S. australis* | 1 | Mt Sutton | G | A | C |  |  | C |  | G |  | C | A | 2 | G | A | A | T/C | C | C |
| *S. australis* | 2 | Remarkables | G | A | G |  |  | C | 2 | C |  | A | G | | C/G | A | A | A | C | C |
| *S. obelisci* | 2 | Obelisk | G | G | C |  |  | C |  | G |  | C | A |  | C | G | A | C | G | C |
| *S. childi* | 1 | Earnscleugh | G | G | C |  |  | C | 3 | G |  | C | A |  | C | G | A | A | G | A |
| *S. australis* | 4 | Airport/Dunstan Range/Lindis/Lindis | G | G | C |  |  | C |  | G |  | C | A |  | C | G | A | A | C | A |
| *S. childi* | 1 | Earnscleugh | G | G | C |  |  | C |  | G |  | C | A |  | C | G | A | A | G/C | A |
| *S. obelisci* | 1 | Obelisk | G | G | C |  |  | C |  | G |  | C | A |  | C | G | A | C | G | C |
| *S. childi*, *S. australis* | 2 | Airport/Lower ManorBurn | G | G | C |  |  | G |  | G |  | C | A |  | C | G | A | A | C | A |
| *S. australis* | 1 | Conroy Dam | G | G | C |  |  | HYB |  | G |  | C | A |  | C | G | A | A | C | A |
| *S. australis* | 1 | Airport | G | G | C |  |  | S |  | G |  | C | A |  | C | G | A | A | G/C | A |
| *S. australis* | 1 | Raggedy Range | G | G | C/G |  |  | HYB |  | G |  | C | A |  | C | G | A | A | C | A |
| *S. childi* | 1 | Crawford Rd | G | G/A | C |  |  | HYB |  | G | 9 | C | A |  | C | G | A | C/A | G | C/A |
| *S. childi* | 1 | Earnscleugh | G | G/A | C |  |  | HYB |  | G |  | C | A |  | C | G | A | A | C/G | A |
| *S. childi* | 1 | ManorBurn | G | G/A | C |  |  | HYB |  | G |  | C | A |  | C | G | A | A/C | G/C | SHORT |
| *S. australis* | 1 | Airport | G/A | G/A | C |  |  | HYB |  | G |  | C | A |  | C | G | A | A/C | G/C | A |
| *S. childi* | 1 | Crawford Hills | G/A | A | C |  | 3 | HYB | 3 | G |  | C | A |  | C | G | A | A | C/G | A |
| *S. childi*, *S. australis* | 3 | Graveyard Gully/Graveyard Gully/Airport | G/A | G/A | C |  | 3 | HYB |  | G | 9 | C | A |  | C | G | A | A | C | A |
| *S. childi* | 1 | Crawford Rd | G/A | G/A | C |  | 3 | HYB |  | G |  | C | A |  | C | G | A | A | G/C | A |
| *S. childi* | 1 | Little Valley | G/A | G/A | C |  |  | HYB |  | G | 9 | C | A |  | C | G | A | A | C | A |
| *S. childi*, *S. australis* | 2 | Airport/Manor Burn | G/A | G/A | C |  |  | HYB |  | G |  | C | A |  | C | G | A | A | C | A |
| *S. childi* | 1 | Manor Burn | G/A | G/A | C |  |  | HYB |  | G |  | C | A |  | C | G | A | A | C/G | A/C |
| *S. childi*, *S. australis* | 2 | Little Valley | G/A | G/A | C |  |  | HYB |  | G |  | C | A |  | C | G | A | A | G/C | A |

**References**

Rozen S, Skaletsky HJ (2000) *Primer3 on the WWW for general users and for biologist programmers*. Humana Press, Totowa, NJ.
